# Depression, anxiety, lower sleep quality and social support in square cabin hospitals during Shanghai’s COVID-19 lockdown, China

**DOI:** 10.3389/fpsyt.2024.1339774

**Published:** 2024-02-05

**Authors:** Li Quan, Shuyu Xu, Hao Xu, Feng Chen, Shengyong Wu, Jiaqi Zhu, Suxuan Liu, Tong Su

**Affiliations:** ^1^Department of Cardiology, Shanghai Eastern Hepatobiliary Surgery Hospital, Naval Medical University, Shanghai, China; ^2^Faculty of Psychology, Naval Medical University, Shanghai, China; ^3^Department of Infectious Diseases, the First Affiliated Hospital (Changhai Hospital) of Naval Medical University, Shanghai, China; ^4^Department of Cardiology, the First Affiliated Hospital (Changhai Hospital) of Naval Medical University, Shanghai, China; ^5^Department of Military Health Statistics, Naval Medical University, Shanghai, China

**Keywords:** square cabin hospitals, depression, anxiety, sleep, social support, isolation wards

## Abstract

**Objectives:**

To investigate and compare the associated factors of depression, anxiety, and other psychological differences between patients with Corona Virus Disease 2019 quarantined in square cabin hospitals (SCH) and isolation wards (IW) in China.

**Methods:**

Cluster sampling method was performed during Shanghai’s Two-Month Lockdown in 2022. Hospital Anxiety and Depression Scale Depression subscale (HADS-D), 7-tiem Generalized Anxiety Disorder Scale (GAD-7), Pittsburgh sleep quality index (PSQI), and Perceived Social Support Scale (PSSS) were used to investigate psychological differences.

**Results:**

The HADS-D and GAD-7 scores of SCH patients were significantly higher than those in IW (p < 0.001; p = 0.0295). Sleep latency (SCH-IW = -3.76, p < 0.001), sleep duration (SCH-IW = -2.22, p < 0.05), habitual sleep efficiency (SCH-IW = -4.11, p < 0.001), sleep disturbance (SCH-IW = -3.59, p < 0.001) and use of sleep medication (SCH-IW = -5.18, p < 0.001) of SCH patients were significantly worse. Depression was the main emotional problem of quarantined patients. Patients in SCH had lower social support. Sleep disorders and the lowest oxygen saturation ≤ 93% were risk factors for depression, while social support and child status were protective factors. Myalgia and constipation were risk factors for anxiety, while marital status was the protective factor.

**Conclusion:**

Patients quarantined in SCH had higher risks of depression and anxiety, lower sleep quality and social support. Somatic discomfort and sleep disorders exacerbated depression and anxiety, which could be ameliorated by social support and taken into consideration in future SCH construction.

## 1 Introduction

Infectious disease epidemics pose significant threats to public health. Given that droplets are the primary mode of transmission for Corona Virus Disease 2019 (COVID-19) and its high transmissibility, it is essential to address not only decontamination and personal protection but also the issue of isolation wards (IW) (1, 2). COVID-19 patients who cannot be cared for in their home location should be cohorted in the IW (3). IW prevents the spread of infectious agents and offers advantages such as a robust infection management system, streamlined policies and procedures, and professional training for medical practitioners (4, 5). However, the capacity of IW is often limited, leading to significant psychological distress among patients, including depression, anxiety, and insomnia (6, 7). Several measures have been implemented to alleviate patients’ psychological distress in IW, including transforming common wards, constructing temporary containers to expand IW’s capacity, and enhancing equipment such as the network system and wireless access points (8, 9).

The global spread of COVID-19 highlighted the insufficient treatment capacity of IW, demanding an immediate increase in surge capacity (8, 9). To address the significantly increased medical pressure, the central government of China introduced mobile emergency hospitals, also known as square cabin hospitals (SCH), to prevent COVID-19 and other rapidly spreading infectious diseases (10, 11). As a cost-effective and easy-to-build modular medical facility, SCH provides essential functions such as isolation, triage, basic medical services, regular monitoring, and rapid diagnosis. Numerous studies have shown that SCH can increase patient admission rates, contain epidemics, and reduce the rate of mild and moderate cases progressing to severe and critical cases, effectively slowing down the spread of COVID-19 (12–15).

With limited environmental conditions and a high patient volume, SCH primarily concentrates on enhancing medical care and physical therapy while overlooking the influence of environmental factors on patients’ psychological and physiological well-being. Few studies have examined the psychological status of patients isolated in SCH and compared it with those in IW. Therefore, this study aimed to survey and compare the psychological status of patients who were quarantined in IW and SCH during Shanghai’s Two-Month Lockdown in 2022. We also explored the factors that affect the psychological status of patients, aiming to provide evidence for SCH construction in the future.

## 2 Methods

### 2.1 Participants

**Figure 1:**
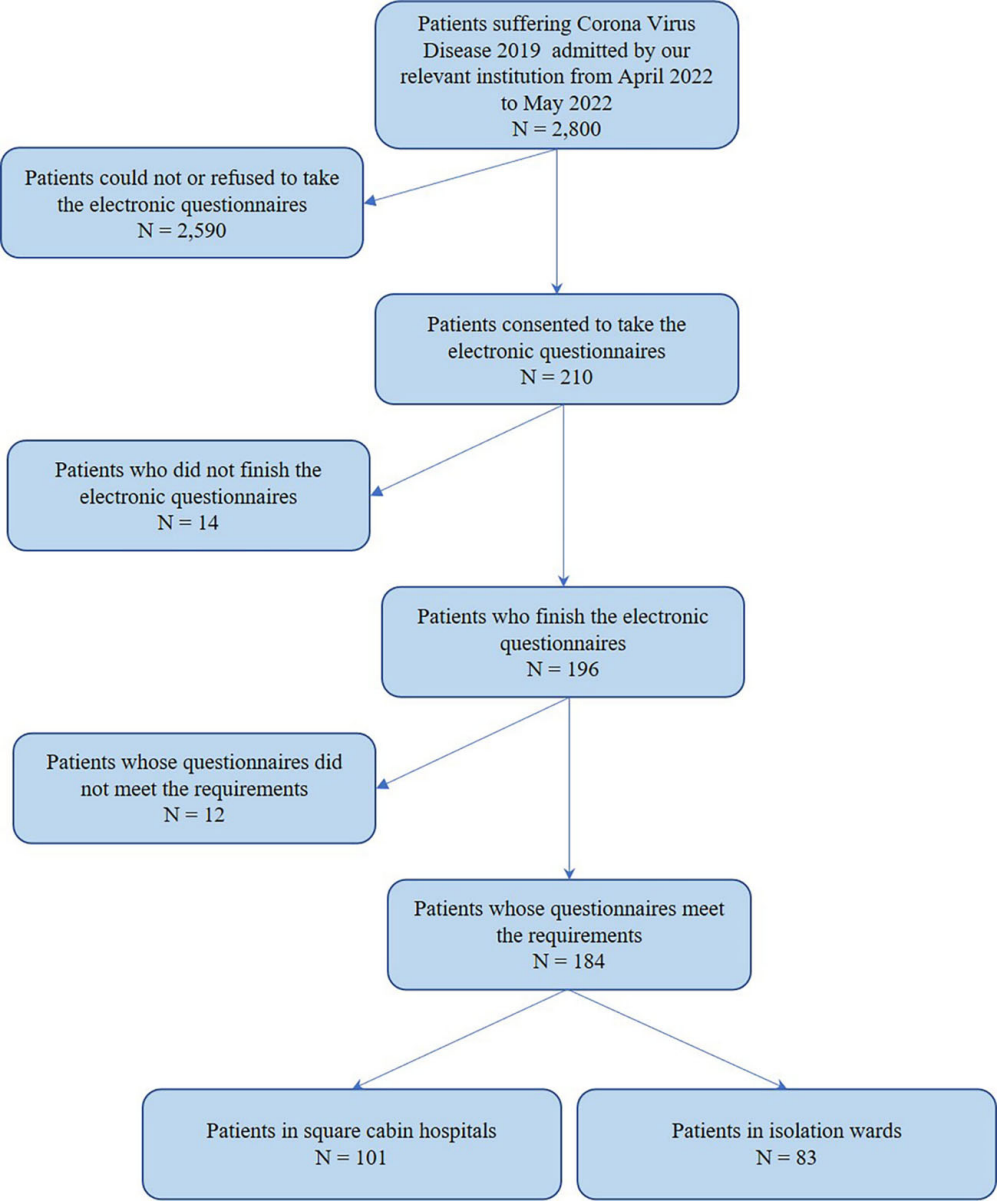
The flow chart of research design.

**Figure 2:**
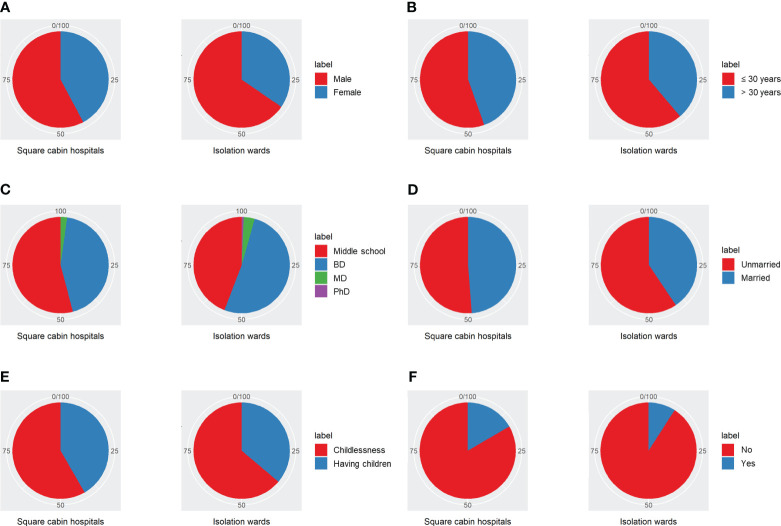
Differences between patients quarantined in SCH and IW. **(A)** Gender of participants. **(B)** Age of participants. **(C)** Education level of participants. **(D)** Marital status of participants. **(E)** Child status of participants. **(F)** Family members infected situation of participants. BD, Bachelor Degree; MD, Master’s Degree; PhD, Doctor of Philosophy.

From April 2022 to May 2022, COVID-19 patients treated in IW and SCH, Shanghai, were invited to finish electronic questionnaires. The cluster sampling method was performed. A total of 184 valid questionnaires were collected, including 101 patients in SCH and 83 patients in IW. The flow chart of this research design is shown in **Figure 1**, and detailed information of participants is shown in **Table 1**; **Figure 2**. This study was approved by the Institutional Ethical Committee of Changhai Hospital (CHEC2022-063) and was performed as a part of a clinical trial (ChiCTR2200059734).

**Table 1:** The detailed information of participants.

Items	Categories	Raw data					Propensity Score Weighting				
		Total, n (%)	SCH, n (%)	IW, n (%)	p value	SMD	Total, n (%)	SCH, n (%)	IW, n (%)	p value	SMD
Gender	Male	94 (51.09)	29 (28.71)	65 (78.31)	<.0001	0.50	208 (60.87)	118 (57.82)	90 (65.42)	0.1578	0.08
	Female	90 (48.91)	72 (71.29)	18 (21.69)			134 (39.13)	86 (42.18)	48 (34.58)		
Age	≤30 years	104 (56.52)	45 (44.55)	59 (71.08)	0.0003	0.27	198 (57.77)	113 (55.46)	84 (61.21)	0.2904	0.06
	>30 years	80 (43.48)	56 (55.45)	24 (28.92)			145 (42.23)	91 (44.54)	53 (38.79)		
Education level	Middle school	114 (61.96)	75 (74.26)	39 (46.99)	0.0002	0.27	172 (50.13)	111 (54.13)	61 (44.18)	0.0393	0.10
	BD	63 (34.24)	23 (22.77)	40 (48.19)		0.25	160 (46.82)	89 (43.71)	71 (51.44)		0.08
	MD	6 (3.26)	3 (2.97)	3 (3.61)		0.01	9 (2.77)	4 (2.17)	5 (3.66)		0.01
	PhD	1 (0.54)	0 (0.00)	1 (1.20)		0.01	1 (0.29)	0 (0.00)	1 (0.73)		0.01
Only child in one’s family	Yes	53 (28.80)	26 (25.74)	27 (32.53)	0.3117	0.07	101 (29.44)	61 (29.97)	39 (28.65)	0.7926	0.01
	No	131 (71.20)	75 (74.26)	56 (67.47)			241 (70.56)	143 (70.03)	98 (71.35)		
Marital status	Unmarried	94 (51.09)	36 (35.64)	58 (69.88)	<.0001	0.34	187 (54.51)	105 (51.19)	82 (59.44)	0.1331	0.08
	Married	90 (48.91)	65 (64.36)	25 (30.12)			156 (45.49)	100 (48.81)	56 (40.56)		
Child status	Childlessness	101 (54.89)	40 (39.60)	61 (73.49)	<.0001	0.34	207 (60.55)	119 (58.32)	88 (63.85)	0.3043	0.06
	Having children	83 (45.11)	61 (60.40)	22 (26.51)			135 (39.45)	85 (41.68)	50 (36.15)		
Family members infected	No	158 (85.87)	82 (81.19)	76 (91.57)	0.0443	0.10	296 (86.39)	170 (83.35)	125 (90.90)	0.0459	0.08
	Yes	26 (14.13)	19 (18.81)	7 (8.43)			47 (13.61)	34 (16.65)	13 (9.10)		
Lowest oxygen saturation ≤93%	No	130 (70.65)	60 (59.41)	70 (84.34)	0.0002	0.25	271 (79.13)	155 (75.87)	116 (83.97)	0.0705	0.08
	Yes	54 (29.35)	41 (40.59)	13 (15.66)			71 (20.87)	49 (24.13)	22 (16.03)		
Fever (≥37.3°C)	No	129 (70.11)	86 (85.15)	43 (51.81)	<.0001	0.33	210 (61.44)	125 (61.29)	85 (61.67)	0.9430	<0.01
	Yes	55 (29.89)	15 (14.85)	40 (48.19)			132 (38.56)	79 (38.71)	53 (38.33)		
Cough	No	57 (30.98)	36 (35.64)	21 (25.30)	0.1311	0.10	102 (29.81)	57 (28.00)	45 (32.50)	0.3721	0.05
	Yes	127 (69.02)	65 (64.36)	62 (74.70)			240 (70.19)	147 (72.00)	93 (67.50)		
Expectoration	No	103 (55.98)	53 (52.48)	50 (60.24)	0.2910	0.08	206 (60.29)	116 (56.68)	90 (65.63)	0.0970	0.09
	Yes	81 (44.02)	48 (47.52)	33 (39.76)			136 (39.71)	89 (43.32)	47 (34.37)		
Tachypnea	No	174 (94.57)	94 (93.07)	80 (96.39)	0.3235	0.03	308 (89.86)	177 (86.56)	130 (94.77)	0.0136	0.08
	Yes	10 (5.43)	7 (6.93)	3 (3.61)			35 (10.14)	27 (13.44)	7 (5.23)		
Fatigue	No	145 (78.80)	79 (78.22)	66 (79.52)	0.8300	0.01	232 (67.68)	134 (65.41)	98 (71.05)	0.2738	0.06
	Yes	39 (21.20)	22 (21.78)	17 (20.48)			111 (32.32)	71 (34.59)	40 (28.95)		
Chest distress	No	169 (91.85)	92 (91.09)	77 (92.77)	0.6782	0.02	304 (88.71)	175 (85.59)	129 (93.34)	0.0263	0.08
	Yes	15 (8.15)	9 (8.91)	6 (7.23)			39 (11.29)	29 (14.41)	9 (6.66)		
Myalgia	No	144 (78.26)	83 (82.18)	61 (73.49)	0.1553	0.09	239 (69.83)	135 (66.23)	103 (75.16)	0.0778	0.09
	Yes	40 (21.74)	18 (17.82)	22 (26.51)			103 (30.17)	69 (33.77)	34 (24.84)		
Palpitate	No	177 (96.20)	98 (97.03)	79 (95.18)	0.5142	0.02	334 (97.64)	201 (98.27)	133 (96.71)	0.3508	0.02
	Yes	7 (3.80)	3 (2.97)	4 (4.82)			8 (2.36)	4 (1.73)	5 (3.29)		
Pharyngalgia	No	117 (63.59)	62 (61.39)	55 (66.27)	0.4937	0.05	216 (63.23)	131 (64.13)	85 (61.91)	0.6760	0.02
	Yes	67 (36.41)	39 (38.61)	28 (33.73)			126 (36.77)	73 (35.87)	52 (38.09)		
Heterosmia	No	175 (95.11)	96 (95.05)	79 (95.18)	0.9672	<0.01	309 (90.17)	179 (87.46)	130 (94.20)	0.0399	0.07
	Yes	9 (4.89)	5 (4.95)	4 (4.82)			34 (9.83)	26 (12.54)	8 (5.80)		
Diarrhea	No	163 (88.59)	90 (89.11)	73 (87.95)	0.8060	0.01	298 (87.14)	178 (86.97)	120 (87.41)	0.9052	<0.01
	Yes	21 (11.41)	11 (10.89)	10 (12.05)			44 (12.86)	27 (13.03)	17 (12.59)		
Constipation	No	174 (94.57)	92 (91.09)	82 (98.80)	0.0218	0.08	331 (96.84)	195 (95.42)	136 (98.95)	0.0668	0.04
	Yes	10 (5.43)	9 (8.91)	1 (1.20)			11 (3.16)	9 (4.58)	1 (1.05)		

SCH, square cabin hospitals; IW, isolation wards; BD, Bachelor Degree; MD, Master’s Degree; PhD, Doctor of Philosophy.

### 2.2 Research tools

#### 2.2.1 General situation investigation

Self-compiling general situation investigation, including age, gender, occupation, work, marriage, and childbirth. Specific items refer to **Table 1**; **Figure 2**.

#### 2.2.2 Hospital anxiety and depression scale - depression subscale

This scale is mainly used for screening patients’ anxiety and depression in general hospitals. The depression subscale was used in this study. HADS-D includes 7 items. The accumulated scores rate the degree of depression: score 0-7, indicating no depression; score 8-10, indicating mild levels of depression; score 11-14, indicating moderate levels of depression; and score 15-21, indicating severe levels of depression (16).

#### 2.2.3 7-item generalized anxiety disorder scale

This scale consists of seven items, with a score ranging from 0 to 21. Among these, 0-4 refers to no generalized anxiety, 5-9 refers to mild generalized anxiety, 10-14 refers to moderate generalized anxiety, and 15-21 refers to severe generalized anxiety. This scale is sensitive and specific for the diagnosis and efficacy evaluation of anxiety. It is widely used in hospitals and medical institutions at home and abroad (17).

#### 2.2.4 Pittsburgh sleep quality index

This scale, comprising 19 self-evaluation items, is frequently utilized to assess the sleep quality of the general population. It includes 7 dimensions (subjective sleep quality, sleep latency, sleep duration, sleep efficiency, sleep disturbances, use of sleeping medications, and daytime dysfunction). Each dimension is scored from 0 to 3. The cumulative score of each component is the total PSQI score, ranging from 0 to 21, and those with higher scores have poorer sleep quality (18). There is good internal consistency among the seven dimensions of PSQI: Cronbach α = 0.8420, and the test-retest reliability of the cumulative score is 0.8092 (19). This study set the PSQI total score to 7 as the separatrix to distinguish between those with poor sleep and good sleep.

#### 2.2.5 Perceived social support scale

This scale is mainly used to evaluate the perceived support level of individuals at three levels: family, friends, and other ways. It adopts a 7-level scoring method (1-7 points), with a total score of 12-36 indicating a low support level, 37-60 indicating moderate support level, and 61-84 indicating a high support level. The higher the total score, the higher the individual perceived social support (20).

### 2.3 Statistical methods

Continuous variables were expressed as means and standard deviations or median and interquartile ranges according to data distributions, and categorical variables were expressed as counts and percentages. Continuous, categorical and ordinal variables were analyzed with Student’s t-test, Pearson chi-square test or Cochran-Mantel-Haenszel test, respectively.

Given the differences between groups in the baseline characteristics, we used propensity score weighting (PSW), which was weighted by the inverse probability of treatment weighting (IPTW) method, to reduce the indication bias of treatment allocation. The probability of each patient accessing various medical institutions was estimated by a multivariable logistic regression analysis based on their baseline covariates, including the demographic data, patient symptoms, and medical history of patients (**Table 1**). Variables included in the propensity scores were prespecified before the outcome analysis and included all baseline covariates described above (21). Since the data were processed by propensity score weighting, according to the statistical requirements, adequacy matching for no significant imbalance of each baseline covariate was assessed by standardized mean differences (SMDs). SMD less than 0.1 implied a balanced characteristic between the groups (22).

Univariable and multivariable analyses were performed to evaluate the association between all risk factors and adverse outcomes, including depression and anxiety. Step-wise backward proceeding multiple linear regression models were used to develop the final adjusted models (sls = 0.10, sle = 0.05). Variables eligible for multivariable analysis included all risk factors.

All tests were two-tailed, and p < 0.05 was considered significant unless otherwise specified. Statistical analysis was performed using SAS version 9.4 (SAS Institute Inc) and R version 4.0.4 (R Foundation for Statistical Computing).

## 3 Results

### 3.1 Differences between patients quarantined in SCH and IW

#### 3.1.1 Depression and anxiety

**Figure 3:**
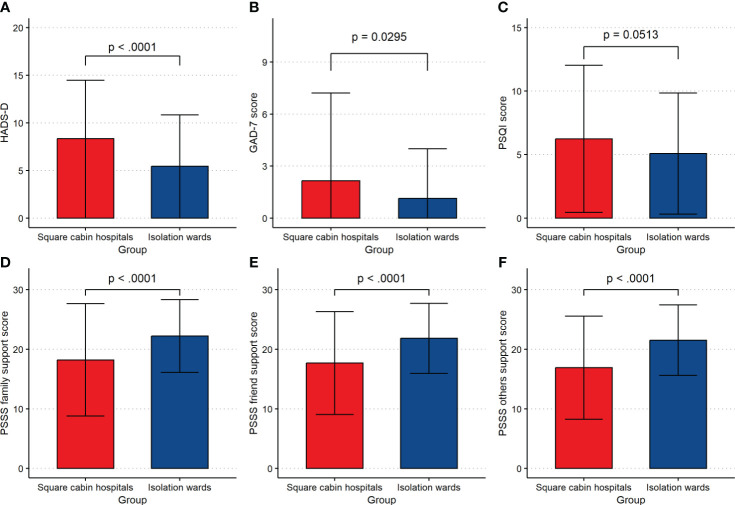
The detailed information of participants. **(A)** HADS-D score. **(B)** GAD-7 score. **(C)** PSQI total score. **(D)** PSSS family support score. **(E)** PSSS friend support score. **(F)** PSSS others support score. HADS-D, Hospital Anxiety and Depression Scale - Depression score; GAD-7, 7-tiem Generalized Anxiety Disorder Scale; PSQI, Pittsburgh Sleep Quality Index; PSSS, Perceived Social Support Scale.

After propensity score weighting, the average HADS-D score of patients quarantined in SCH was significantly higher than those quarantined in IW (SCH vs. IW: 8.33 ± 6.14 vs. 5.43 ± 5.39, p < 0.001), which reached a mild depression. Meanwhile, the GAD-7 scores of patients quarantined in SCH were significantly higher than those quarantined in IW (SCH vs. IW: 2.16 ± 5.07 vs. 1.13 ± 2.87, p = 0.0295), but they all did not meet the criteria for generalized anxiety (**Table 2**; **Figures 3A, B**). The results indicate that patients quarantined in SCH have a higher risk of depression and anxiety than those quarantined in IW.

**Table 2:** Differences between patients quarantined in SCH and IW.

Items	SCH	IW	p value*
HADS-D, mean ± SD	8.33 ± 6.14	5.43 ± 5.39	<.0001
GAD-7, mean ± SD	2.16 ± 5.07	1.13 ± 2.87	0.0295
PSSS, mean ± SD			
family support, mean ± SD	18.21 ± 9.44	22.24 ± 6.12	<.0001
friend support, mean ± SD	17.69 ± 8.65	21.83 ± 5.87	<.0001
others support, mean ± SD	16.91 ± 8.67	21.52 ± 5.93	<.0001
PSQI			
total score, mean ± SD	6.23 ± 5.79	5.07 ± 4.77	0.0513
subjective sleep quality, n (%)			
level0	34(16.55)	35(25.19)	0.5547
level1	112(54.60)	65(47.01)	
level2	44(21.49)	22(15.99)	
level3	15(7.36)	16(11.81)	
sleep latency, n (%)			
level0	47(23.13)	57(41.41)	0.0002
level1	67(32.64)	50(35.98)	
level2	80(38.96)	19(13.61)	
level3	11(5.26)	12(9.00)	
sleep duration, n (%)			
level0	114(55.85)	99(72.00)	0.0261
level1	43(21.25)	12(8.37)	
level2	44(21.69)	21(15.36)	
level3	2(1.20)	6(4.27)	
habitual sleep efficiency, n (%)			
level0	112(54.78)	101(73.12)	<.0001
level1	28(13.67)	22(15.99)	
level2	26(12.62)	6(4.64)	
level3	39(18.93)	9(6.25)	
sleep disturbance, n (%)			
level0	26(12.85)	37(26.54)	0.0003
level1	143(69.72)	89(64.28)	
level2	36(17.43)	13(9.17)	
use of sleep medication, n (%)			
level0	201(98.53)	113(82.40)	<.0001
level1	2(0.98)	20(14.83)	
level2	1(0.49)	3(2.05)	
level3	0(0.00)	1(0.73)	
daytime dysfunction, n (%)			
level0	93(45.69)	56(40.87)	0.8716
level1	42(20.46)	32(23.06)	
level2	32(15.61)	39(28.45)	
level3	37(18.25)	10(7.62)	

*SCH vs. IW, Student’s t-test or Pearson chi-square test after propensity score weighting (PSW); SCH, square cabin hospitals; IW, isolation wards; HADS-D, Hospital Anxiety and Depression Scale - Depression score; GAD-7, 7-tiem Generalized Anxiety Disorder Scale; PSSS, Perceived Social Support Scale; PSQI, Pittsburgh Sleep Quality Index.

#### 3.1.2 Sleep quality

There were significant differences in sleep latency (p = 0.0002), sleep duration (p = 0.0261), habitual sleep efficiency (p < 0.0001), sleep disturbance (p = 0.0003), use of sleep medication (p < 0.0001) between patients quarantined in SCH and those in IW (**Table 2**; **Figure 3C**). The results indicate that patients quarantined in SCH have relatively poor sleep quality.

#### 3.1.3 Social support

Patients quarantined in SCH had lower scores than those quarantined in IW in family support (SCH vs. IW: 18.21 ± 9.44 vs. 22.24 ± 6.12, p < 0.001), friend support (SCH vs. IW: 17.69 ± 8.65 vs. 21.83 ± 5.87, p < 0.001), and other support (SCH vs. IW: 16.91 ± 8.67 vs. 21.52 ± 5.93, p < 0.001), which indicated patients quarantined in SCH felt less social support than those quarantined in IW (**Table 2**; **Figures 3D–F**).

### 3.2 Factors associated with depression and anxiety among patients quarantined in SCH

Risk factor analysis was conducted using the HADS-D score or GAD-7 score as the dependent variable and demographic variables, social support, sleep disorders, and somatic symptoms as independent variables (**Tables 3**, **4**).

**Table 3:** Statistically significant factors associated with depression for patients quarantined in SCH.

Variables	Univariate β value	Univariate p value	Multivariate β value	Multivariate p value
Social support	-3.27 (-4.28 to -2.26)	<.0001	-2.98 (-3.99 to -1.97)	<.0001
Sleep disorders	1.47 (-0.21 to 3.15)	0.0858	2.03 (0.75 to 3.31)	0.0022
Child status	-2.70 (-4.22 to -1.17)	0.0007	-1.70 (-3.06 to -0.34)	0.0149
Lowest oxygen saturation ≤ 93%	2.15 (0.59 to 3.70)	0.0073	1.92 (0.63 to 3.22)	0.0040

SCH, square cabin hospitals.

**Table 4:** Statistically significant factors associated with anxiety for patients quarantined in SCH.

Variables	Univariate β value	Univariate p value	Multivariate β value	Multivariate p value
Marital status	-1.05 (-2.15 to 0.06)	0.0626	-1.28 (-2.30 to -0.27)	0.0140
Myalgia	2.65 (1.34 to 3.95)	0.0001	2.63 (1.32 to 3.94)	0.0001
Constipation	2.02 (0.18 to 3.86)	0.0320	1.78 (0.02 to 3.54)	0.0469

SCH, square cabin hospitals.

#### 3.2.1 Factors associated with depression

Univariate analysis showed age > 30 years (β = -1.87, p = 0.0187), social support (β = -3.27, p < 0.0001), marital status (β = -2.80, p = 0.0006), and child status (β = -2.70, p = 0.0007) were the protective factors of depression, while lowest oxygen saturation ≤ 93% (β = 2.15, p = 0.0073) was the risk factor of depression.

Multivariate analysis showed social support (β = -2.98, 95%CI: -3.99 to -1.97, p < 0.0001) and child status (β = -1.70, 95%CI: -3.06 to -0.34, p = 0.0149) were the protective factors of depression, while sleep disorders (β = 2.03, 95%CI: 0.75 to 3.31, p = 0.0022) and lowest oxygen saturation ≤ 93% (β = 1.92, 95%CI: 0.63 to 3.22, p = 0.0040) were the risk factors of depression (**Tables 3**, **5**). The results indicate that patients with less social support, childlessness, sleep disorders, and lowest oxygen saturation ≤ 93% are more likely to be depressive.

**Table 5:** Factors associated with depression and anxiety.

	Depression	Anxiety
Protective factors	Social support Child status	Marital status
Risk factors	Sleep disorders Lowest oxygen saturation ≤ 93%	Myalgia Constipation

#### 3.2.2 Factors associated with anxiety

Univariate analysis showed education level, sleep disorders, cough, tachypnea, myalgia, heterosmia, and constipation were the risk factors of anxiety (p < 0.05) (**Table 4**). Multivariate analysis showed myalgia (β = 2.63, 95%CI: 1.32 to 3.94, p = 0.0001) and constipation (β = 1.78, 95%CI: 0.02 to 3.54, p = 0.0469) were the risk factors of anxiety, while marital status (β = -1.28, 95%CI: -2.30 to -0.27, p = 0.0140) was the protective factor of anxiety (**Tables 4**, **5**).

## 4 Discussion

### 4.1 Patients quarantined in SCH had relatively poor psychological status and sleep quality

Regarding emotional state, those recruited patients were generally at a high level of depressive mood. After comparing the depression levels of patients under different treatment environments, we found that patients quarantined in SCH had higher levels of depression and anxiety than those quarantined in IW. Compared with IW, Patients in SCH had already reached a mild depressive state.

Our study also found another exciting result: anxiety was not the main emotional problem of COVID-19 patients in SCH or IW, respectively, whether from the overall perspective or the perspective of patients. This is a good sign compared with previous studies (23, 24), which to some extent, reflects that patients were more optimistic about their recovery. Under regular epidemic prevention and control, individuals had a relatively complete understanding of the pathogenesis, routes, and methods of transmission, prevention, and management of COVID-19, and the impact and pressure of viral infection had been reduced.

Our study demonstrated that the sleep quality of patients quarantined in SCH was relatively poor. Furthermore, patients quarantined in SCH had longer sleep latency, poorer sleep duration, lower habitual sleep efficiency, and more severe sleep disturbance. Compared to IW, SCHs have stronger medical capabilities and can treat a large number of patients in a short time. However, problems such as poor airtightness, poor sound insulation, and strong vibration also affect sleep. As a state of rest, sleep is an essential factor in recovery. Insufficient sleep may hamper immune function, decrease energy levels, emotional and cognitive abilities, and increase the risk of trauma.

In contrast, healthy sleep significantly contributes to the recovery and maintenance of molecules and cells, brain growth, immune system balance, and the recovery of diseases and injuries. Patients usually suffer from pain, dyspnea, and other sleep-related disorders compared to healthy people. Therefore, improving the quality of the sleep environment and promoting sleep is of great significance for the rehabilitation of patients.

Compared to patients quarantined in IW, patients in SCH subjectively perceived lower levels of social support regarding family, friends, and other support. This may because SCH is not a traditional hospital ward environment. Although the overall area of SCHs is large, the average living space of patients is relatively minor when there are many patients. They received less attention from doctors and nurses than in IW, which may be one of the reasons why patients in SCH perceived less social support subjectively (25, 26).

### 4.2 Somatic discomfort and sleep disorders are risk factors for patients’ psychological status

The risk factors analysis showed that somatic symptoms such as lowest oxygen saturation ≤ 93%, myalgia, and constipation were risk factors for the patient’s psychological health. The more obvious the somatic symptoms, the more severe patients present with depression and anxiety. A large number of studies worldwide have reported the coexistence of somatic symptoms and emotional problems in patients (27, 28). Some studies also showed that patients with psychological diseases were accompanied by anxiety and depression (29). Al-Jassas et al. also proved COVID-19 infected patients accompanied by lung lesions and low SpO_2_ might exert neuropsychiatric symptoms, due to the activation of immune-inflammatory pathways (30). Studies also showed that depressed people had a higher risk of constipation and vice versa, which might due to inflammation, immune abnormalities, energy metabolism, gut microbiota, and metabolites, like choline, betaine, and glycine (31–34). In recent years, a large number of research on medical models has been conducted globally to improve the treatment effectiveness and patients’ quality of life. The result demonstrated that adopting a patient-centered notion, which requires healthcare systems to prioritize patient needs and feelings from a doctor-patient relationship based on paternalism and physician authority, could get a better therapeutic effect. This approach places specialists, doctors, and patients on the same level, allowing healthcare providers to better understand patients’ expectations, feelings, and social backgrounds toward their diseases. These issues will be taken into therapy while being considered and discussed.

In addition, the results of our study also showed that sleep disorders were risk factors for depression and anxiety among patients in SCH. The poorer the sleep quality of patients, the higher their feelings of depression and anxiety emotions. Sleep problems are often related to the main diagnostic criteria for many mental disorders. The core symptoms of depression diagnosis include sleep interruption and hypersomnia, and the diagnosis of anxiety disorders also includes sleep problems. The circadian rhythm can explain the relationship between sleep and psychological status. The circadian rhythm change will affect the cortisol level, which may put individuals in a state of imbalance (35). During the epidemic, the circadian rhythm was significantly affected due to changes in people’s daily life and social life (36), and more sleep problems were reported. At the same time, there was a clear correlation between the decline in sleep quality and the negative emotional state of individuals during the epidemic (37–39). Some studies also proved that depression (40) and anxiety (41) symptoms could be ameliorated by improving sleep problems.

### 4.3 Future construction of SCH during pandemics

Our study found that in terms of social support, compared to patients quarantined in IW, patients in SCH had a lower level of support from family, friends, and others. Patients quarantined in SCH had a higher level of depression than those in IW, which could potentially be attributed to their relatively low level of social support. Social support is beneficial for patients’ psychological health, and it is shown social support can help alleviate patients’ anxiety and insomnia symptoms when facing stress (42), and can be an indicator to predict psychological status (43).

During the epidemic, to minimize the spread of the virus, most countries made maintaining social distancing one of the main strategies for epidemic prevention. This policy interrupted daily activities, limited mobility, and reduced communication among most people, especially those receiving treatment. Furthermore, social support from family and friends also decreased. Low social support levels were more likely to lead to depression and anxiety symptoms in those under stress (44). During the COVID-19 pandemic 2020, a study was implemented to explore the impact of social support on the psychological health of Chinese adolescents, which showed that the prevalence of psychological problems was higher among adolescents with low and medium levels of social support (45).

Therefore, in the future construction of SCH, consideration should also be given to increase humanistic construction related to social support, such as increasing recreation facilities, video calling equipment, or safe visiting areas for healthy family members. For those who are prone to depression or anxiety, like single, divorced, or childless individuals, support from medical practitioners, mutual help and collaboration among patients need further increase, so as to narrow the psychological distance of patients (46). Some group activities could be performed for mild patients in SCH, like a talent show, disease prevention and treatment lecture, or Baduanjin exercise, a traditional Chinese mind-body exercise (47). When a patient’s family members suffer from COVID-19, they can be quarantined in the same place, if possible. Thus, more high social support environments, such as parent-child wards, family wards (with family care), and community wards should be established (48). In addition to medication treatment, the proportion of non-pharmacological intervention, such as on-the-spot and long-distance psychological counseling, as well as other various intervention measures, can also be added to help COVID-19 patients recover (49–51).

We also found sleep disorders and the lowest oxygen saturation ≤ 93% were risk factors for depression. Sleep quality of SCH patients was significantly worse than IW patients. As the most prominent symptom in depressive patients, sleep disturbance plays an important role in the development of depression, and patients with sleep disorders are more prone to depression (52–54). To improve the psychological status of patients, sleep quality improvement also needs to be considered, which includes improving airtightness, controlling light and noise, enhancing sound insulation, reducing vibration, strengthening privacy protection, adjusting patients’ circadian rhythm, providing eye masks, earplugs, shading curtains, and playing light music for patients before bedtime (55–57). As researches also showed COVID-19 patients with low oxygen saturation were often accompanied by depression (30, 58), blood-oxygen monitors and adequate oxygen supply were needed in SCH construction. Similar improvement can also be applied in IW, or other emergency or disaster preparedness situations.

There are still some limitations in our study. It was an observational study. Because the study was conducted during the lockdown period, we could not intervene in patients’ sleep to further investigate psychological status improvement. Additionally, we could not exclude some potential confounding factors. Moreover, the sample size of this study is also relatively small. Further studies need to be carried out in the future.

## 5 Conclusions

The present study demonstrated depression was the main emotional problem of COVID-19 patients during Shanghai’s Two-Month Lockdown in 2022. Compared to IW, patients treated in SCH exhibited higher levels of depression, lower sleep quality, and relatively lower social support. Somatic discomfort and sleep disorders could exacerbate the depression and anxiety of patients in SCH, which could be alleviated by social support. Improving patients’ sleep quality and social support is a feasible way to relieve their depression and anxiety and thus help patients better restore their health. In future hospital construction projects aimed at enhancing the effectiveness of physiotherapy, it is imperative also to prioritize the improvement of patients’ sleep and living conditions. Additionally, providing appropriate social support, coupled with the inclusion of psychological counseling and services, can significantly enhance patients’ psychological well-being. Similar needs of residents should also be considered in other emergency or disaster preparedness situations, like a temporary shelter in earthquake rescue.

## Data availability statement

The raw data supporting the conclusions of this article will be made available by the authors, without undue reservation.

## Ethics statement

The studies involving humans were approved by Institutional Ethical Committee of Changhai Hospital. The studies were conducted in accordance with the local legislation and institutional requirements. Written informed consent for participation in this study was provided by the participants’ legal guardians/next of kin.

## Author contributions

LQ: Writing – original draft. SX: Writing – original draft. HX: Investigation, Writing – original draft. FC: Conceptualization, Writing – original draft. SW: Formal analysis, Methodology, Writing – review & editing. JZ: Investigation, Writing – review & editing. SL: Conceptualization, Funding acquisition, Supervision, Writing – review & editing. TS: Conceptualization, Funding acquisition, Supervision, Writing – review & editing.

## References

[1] Voidarou C Rozos G Stavropoulou E Giorgi E Stefanis C Vakadaris G. COVID-19 on the spectrum: a scoping review of hygienic standards. Front Public Health (2023) 11:1202216. doi: 10.3389/fpubh.2023.1202216 38026326 PMC10646607

[2] Zhang Y Han O Li AG Hou LA Olofsson T Zhang LH. Adaptive wall-based attachment ventilation: A comparative study on its effectiveness in airborne infection isolation rooms with negative pressure. Eng (Beijing) (2022) 8:130–7. doi: 10.1016/j.eng.2020.10.020 PMC7825860 33520328

[3] Liu HY Liu ZJ Wang YX Hu CX Rong R. Distribution of droplets/droplet nuclei from coughing and breathing of patients with different postures in a hospital isolation ward. Build Environ (2022) 225:109690. doi: 10.1016/j.buildenv.2022.109690 36246843 PMC9547661

[4] Zhou Y Bai LX Guo H Guo SW Han XW Yue NJ. SWOT analysis and preliminary study on prevention and control management of temporary integrated isolation ward during COVID-19 outbreak. Front Public Health (2021) 9:558565. doi: 10.3389/fpubh.2021.558565 33791264 PMC8005535

[5] Saravia SA Raynor PC Streifel AJ. A performance assessment of airborne infection isolation rooms. Am J Infect Control (2007) 35:324–31. doi: 10.1016/j.ajic.2006.10.012 17577480

[6] Hu YY Chen YY Zheng YX You CP Tan J Hu L. Factors related to mental health of inpatients with COVID-19 in Wuhan, China. Brain Behav Immun (2020) 89:587–93. doi: 10.1016/j.bbi.2020.07.016 PMC7362867 32681866

[7] Xing LQ Xu L Sun J Wang QX Ge DD Jiang MM. Anxiety and depression in frontline health care workers during the outbreak of Covid-19. Int J Soc Psychiatry (2021) 67:656–63. doi: 10.1177/0020764020968119 33100114

[8] Ganesan S Balasubramanian B Krishnamurthy P Govindan R Mani N. Effects of tele-counseling on reducing anxiety levels of COVID-19 patients in isolation wards: an observational study. Indian J Psychol Med (2023) 45:43–6. doi: 10.1177/02537176221139598 PMC9896101 36778622

[9] Wee LE Fan EMP Heng R Ang SY Chiang JL Tan TT. Construction of a container isolation ward: A rapidly scalable modular approach to expand isolation capacity during the coronavirus disease 2019 (COVID-19) pandemic. Infect Control Hosp Epidemiol (2021) 42:1162–4. doi: 10.1017/ice.2020.1222 PMC7562929 32962768

[10] Zhao S Lin Q Ran J Musa SS Yang G Wang W. Preliminary estimation of the basic reproduction number of novel coronavirus (2019-nCoV) in China, from 2019 to 2020: A data-driven analysis in the early phase of the outbreak. Int J Infect Dis (2020) 92:214–7. doi: 10.1016/j.ijid.2020.01.050 PMC7110798 32007643

[11] Lai CC Shih TP Ko WC Tang HJ Hsueh PR. Severe acute respiratory syndrome coronavirus 2 (SARS-CoV-2) and coronavirus disease-2019 (COVID-19): The epidemic and the challenges. Int J Antimicrob Agents (2020) 55:105924. doi: 10.1016/j.ijantimicag.2020.105924 32081636 PMC7127800

[12] Shang L Xu J Cao B. Fangcang shelter hospitals in COVID-19 pandemic: the practice and its significance. Clin Microbiol Infect (2020) 26:976–8. doi: 10.1016/j.cmi.2020.04.038 PMC7252175 32360781

[13] Li J Yuan P Heffernan J Zheng T Ogden N Sander B. Fangcang shelter hospitals during the COVID-19 epidemic, Wuhan, China. Bull World Health Organ (2020) 98:830–41D. doi: 10.2471/BLT.20.258152 33293743 PMC7716094

[14] Jiang H Song P Wang S Yin S Yin J Zhu C. Quantitative assessment of the effectiveness of joint measures led by Fangcang shelter hospitals in response to COVID-19 epidemic in Wuhan, China. BMC Infect Dis (2021) 21:626. doi: 10.1186/s12879-021-06165-w 34210269 PMC8245925

[15] Li H Lian H Lin J Chen K Lyu Y Chen Y. Mobile cabin hospital compulsory quarantine for mild patients can serve as an alternative treatment for COVID-19: the Chinese experience. Am J Transl Res (2022) 14:3132–42. PMC9185049 35702103

[16] Chung DXY Loo YE Kwan YH Phang JK Woon TH Goh WR. Association of anxiety, depression and resilience with overall health and functioning in axial spondyloarthritis (axSpA): a cross-sectional study. BMJ Open (2023) 13:e071944. doi: 10.1136/bmjopen-2023-071944 PMC10174021 37156581

[17] Garabiles MR Lao CK Yip P Chan EWW Mordeno I Hall BJ. Psychometric validation of PHQ-9 and GAD-7 in filipino migrant domestic workers in macao (SAR), China. J Pers Assess (2020) 102:833–44. doi: 10.1080/00223891.2019.1644343 31361153

[18] Niu SZ Wu Q Ding SL Wu LC Wang L Shi Y. Comparison of three measures for insomnia in ischemic stroke patients: Pittsburgh sleep quality index, insomnia severity index, and Athens insomnia scale. Front Neurol (2023) 14:1118322. doi: 10.3389/fneur.2023.1118322 37712082 PMC10498538

[19] Lu TY Li Y Xia P Zhang GQ Wu DR. Analysis on reliability and validity of the Pittsburgh sleep quality index. Chongqing Med (2014) 43:260–3.

[20] Wang SC Li M Xiang X Guo XJ Peng CL Wang DP. Analysis on the current situation of twin breastfeeding and its influencing factors. Med (Baltimore) (2023) 102:e35161. doi: 10.1097/MD.0000000000035161 PMC10519451 37746974

[21] Bettuzzi T Jourdes A Robineau O Alcaraz I Manda V Molina JM. Ceftriaxone compared with benzylpenicillin in the treatment of neurosyphilis in France: a retrospective multicentre study. Lancet Infect Dis (2021) 21:1441–7. doi: 10.1016/S1473-3099(20)30857-4 34051142

[22] Brito JP Deng YH Ross JS Choi NH Graham DJ Qiang YD. Association between generic-to-generic levothyroxine switching and thyrotropin levels among US adults. JAMA Intern Med (2022) 182:418–25. doi: 10.1001/jamainternmed.2022.0045 PMC8886450 35226058

[23] Abraham A Jithesh A Doraiswamy S Al-Khawaga N Mamtani R Cheema S. Telemental health use in the COVID-19 pandemic: A scoping review and evidence gap mapping. Front Psychiatry (2021) 12:748069. doi: 10.3389/fpsyt.2021.748069 34819885 PMC8606591

[24] Gu Y Zhu Y Xu F Xi J Xu G. Factors associated with mental health outcomes among patients with COVID-19 treated in the Fangcang shelter hospital in China. Asia Pac Psychiatry (2021) 13:e12443. doi: 10.1111/appy.12443 33135397

[25] Zhu W Wang Y Xiao K Zhang H Tian Y Clifford SP. Establishing and managing a temporary coronavirus disease 2019 specialty hospital in wuhan, China. Anesthesiology. (2020) 132:1339–45. doi: 10.1097/ALN.0000000000003299 PMC7155903 32195700

[26] Wu J Shen B Li D Song W Li J Zhang M. Pharmacy services at a temporary COVID-19 hospital in Wuhan, China. Am J Health Syst Pharm (2020) 77:1186–7. doi: 10.1093/ajhp/zxaa160 PMC7314153 32474580

[27] Janzen ML LeComte K Sathananthan G Wang J Kiess M Chakrabarti S. Psychological distress in adults with congenital heart disease over the COVID-19 pandemic. J Am Heart Assoc (2022) 11:e023516. doi: 10.1161/JAHA.121.023516 35470701 PMC9238616

[28] Benedetti F Palladini M D'Orsi G Furlan R Ciceri F Rovere-Querini P. Mood-congruent negative thinking styles and cognitive vulnerability in depressed COVID-19 survivors: A comparison with major depressive disorder. J Affect Disord (2022) 308:554–61. doi: 10.1016/j.jad.2022.04.077 PMC9020513 35460737

[29] Ziadni MS You DS Cramer EM Anderson SR Hettie G Darnall BD. The impact of COVID-19 on patients with chronic pain seeking care at a tertiary pain clinic. Sci Rep (2022) 12:6435. doi: 10.1038/s41598-022-10431-5 35440688 PMC9017421

[30] Al-Jassas HK Al-Hakeim HK Maes M. Intersections between pneumonia, lowered oxygen saturation percentage and immune activation mediate depression, anxiety, and chronic fatigue syndrome-like symptoms due to COVID-19: A nomothetic network approach. J Affect Disord (2022) 297:233–45. doi: 10.1016/j.jad.2021.10.039 PMC8541833 34699853

[31] Adibi P Abdoli M Daghaghzadeh H Keshteli AH Afshar H Roohafza H. Relationship between depression and constipation: results from a large cross-sectional study in adults. Korean J Gastroenterol (2022) 80:77–84. doi: 10.4166/kjg.2022.038 36004635 PMC12285336

[32] Liu XJ Liu HL Wei FX Zhao D Wang YZ Lv M. Fecal metabolomics and network pharmacology reveal the correlations between constipation and depression. J Proteome Res (2021) 20:4771–86. doi: 10.1021/acs.jproteome.1c00435 34524820

[33] Liang JJ Zhao YM Xi Y Xiang CH Yong CT Huo JQ. Association between depression, anxiety symptoms and gut microbiota in chinese elderly with functional constipation. Nutrients. (2022) 14:5013. doi: 10.3390/nu14235013 36501044 PMC9740187

[34] Wang PF Shen X Wang Y Jia XQ. Association between constipation and major depression in adult Americans: evidence from NHANES 2005-2010. Front Psychiatry (2023) 14:1152435. doi: 10.3389/fpsyt.2023.1152435 37654986 PMC10465693

[35] Foster RG. Sleep, circadian rhythms and health. Interface Focus (2020) 10:20190098. doi: 10.1098/rsfs.2019.0098 32382406 PMC7202392

[36] Morin CM Carrier J Bastien C Godbout R Canadian S Circadian N. Sleep and circadian rhythm in response to the COVID-19 pandemic. Can J Public Health (2020) 111:654–7. doi: 10.17269/s41997-020-00382-7 PMC7375451 32700231

[37] Coiro MJ Asraf K Tzischinsky O Hadar-Shoval D Tannous-Haddad L Wolfson AR. Sleep quality and COVID-19-related stress in relation to mental health symptoms among Israeli and U.S. adults. Sleep Health (2021) 7:127–33. doi: 10.1016/j.sleh.2021.02.006 33691986

[38] Osiogo F Shalaby R Adegboyega S Hrabok M Gusnowski A Vuong W. COVID-19 pandemic: demographic and clinical correlates of disturbed sleep among 6,041 Canadians. Int J Psychiatry Clin Pract (2021) 25:164–71. doi: 10.1080/13651501.2021.1881127 33606597

[39] Stanton R To QG Khalesi S Williams SL Alley SJ Thwaite TL. Depression, anxiety and stress during COVID-19: associations with changes in physical activity, sleep, tobacco and alcohol use in Australian adults. Int J Environ Res Public Health (2020) 17:4065. doi: 10.3390/ijerph17114065 32517294 PMC7312903

[40] Freeman D Sheaves B Waite F Harvey AG Harrison PJ. Sleep disturbance and psychiatric disorders. Lancet Psychiatry (2020) 7:628–37. doi: 10.1016/S2215-0366(20)30136-X 32563308

[41] Chellappa SL Aeschbach D. Sleep and anxiety: From mechanisms to interventions. Sleep Med Rev (2022) 61:101583. doi: 10.1016/j.smrv.2021.101583 34979437

[42] Xiao H Zhang Y Kong D Li S Yang N. The effects of social support on sleep quality of medical staff treating patients with coronavirus disease 2019 (COVID-19) in january and february 2020 in China. Med Sci Monit (2020) 26:e923549. doi: 10.12659/MSM.923549 32132521 PMC7075079

[43] Gu Y Hu J Hu Y Wang J. Social supports and mental health: a cross-sectional study on the correlation of self-consistency and congruence in China. BMC Health Serv Res (2016) 16:207. doi: 10.1186/s12913-016-1463-x 27353410 PMC4924263

[44] Guntzviller LM Williamson LD Ratcliff CL. Stress, social support, and mental health among young adult hispanics. Fam Community Health (2020) 43:82–91. doi: 10.1097/FCH.0000000000000224 31764309

[45] Qi M Zhou SJ Guo ZC Zhang LG Min HJ Li XM. The effect of social support on mental health in chinese adolescents during the outbreak of COVID-19. J Adolesc Health (2020) 67:514–8. doi: 10.1016/j.jadohealth.2020.07.001 PMC7395830 32753347

[46] Pomey MP Paquette J Iliescu-Nelea M Vialaron C Mourad R Bouchard K. Accompanying patients in clinical oncology teams: Reported activities and perceived effects. Health Expect (2023) 26:847–57. doi: 10.1111/hex.13710 PMC10010089 36704843

[47] Zhang XB Zhang JL Li MX Yuan YP Sun J. Baduanjin exercise can alleviate anxiety and depression of patients with COVID-19 in Square cabin hospital: A cross-sectional survey. Med (Baltimore) (2021) 100:e26898. doi: 10.1097/MD.0000000000026898 PMC8360425 34397916

[48] Li MC Wu ZM Zhang JP. Sleep status of children infected with familial aggregation Omicron variant in Shanghai in parent-child ward. J Clin Pathological Res (2022) 42:2414–9. doi: 10.1016/j.heliyon.2022.e12151 PMC9791361 36578400

[49] Sun XM Liu WQ Gao YY Qin L Feng H Tan HZ. Comparative effectiveness of non-pharmacological interventions for frailty: a systematic review and network meta-analysis. Age Ageing (2023) 52:afad004. doi: 10.1093/ageing/afad004 36746389

[50] Yu DSF Li PWC Lin RSY Kee F Chiu A Wu W. Effects of non-pharmacological interventions on loneliness among community-dwelling older adults: A systematic review, network meta-analysis, and meta-regression. Int J Nurs Stud (2023) 144:104524. doi: 10.1016/j.ijnurstu.2023.104524 37295285

[51] Mombelli S Bacaro V Curati S Berra F Sforza M Castronovo V. Non-pharmacological and melatonin interventions for pediatric sleep initiation and maintenance problems: A systematic review and network meta-analysis. Sleep Med Rev (2023) 70:101806. doi: 10.1016/j.smrv.2023.101806 37406497

[52] Xiao X Rui YM Jin Y Chen M. Relationship of sleep disorder with neurodegenerative and psychiatric diseases: an updated review. Neurochem Res (2023) 18. doi: 10.1007/s11064-023-04086-5 38108952

[53] Fang H Tu S Sheng JF Shao AW. Depression in sleep disturbance: A review on a bidirectional relationship, mechanisms and treatment. J Cell Mol Med (2019) 23:2324–32. doi: 10.1111/jcmm.14170 PMC6433686 30734486

[54] He ZF Tan WY Ma HL Shuai YX Shan ZJ Zhai JX. Prevalence and factors associated with depression and anxiety among older adults: A large-scale cross-sectional study in China. J Affect Disord (2024) 346:135–43. doi: 10.1016/j.jad.2023.11.022 37949242

[55] Pal J Taywade M Pal R Sethi D. Noise pollution in intensive care unit: A hidden enemy affecting the physical and mental health of patients and caregivers. Noise Health (2022) 24:130–6. doi: 10.4103/nah.nah_79_21 PMC9743307 36124521

[56] Ricketts EJ Joyce DS Rissman AJ Burgess HJ Colwell CS Lack LC. Electric lighting, adolescent sleep and circadian outcomes, and recommendations for improving light health. Sleep Med Rev (2022) 64:101667. doi: 10.1016/j.smrv.2022.101667 36064209 PMC10693907

[57] Herscher M Mikhaylov D Barazani S Sastow D Yeo I Dunn AS. A sleep hygiene intervention to improve sleep quality for hospitalized patients. Jt Comm J Qual Patient Saf (2021) 47:343–6. doi: 10.1016/j.jcjq.2021.02.003 33744173

[58] Al-Hadrawi DS Al-Rubaye HT Almulla AF Al-Hakeim HK Maes M. Lowered oxygen saturation and increased body temperature in acute COVID-19 largely predict chronic fatigue syndrome and affective symptoms due to Long COVID: A precision nomothetic approach. Acta Neuropsychiatr (2023) 35:76–87. doi: 10.1017/neu.2022.21 36134517

